# Docetaxel Inhibits Urethral Stricture Formation, an Initial Study in Rabbit Model

**DOI:** 10.1371/journal.pone.0112097

**Published:** 2014-11-06

**Authors:** Delai Fu, Tie Chong, Hecheng Li, Huibo Zhang, Ziming Wang

**Affiliations:** 1 Department of Urology, the Second Affiliated Hospital, School of Medicine, Xi’an Jiaotong University, Xi’an, Shaanxi, People’s Republic of China; 2 Department of Urology, the Affiliated Hospital of Shaanxi Traditional Chinese Medical Collage, Xianyang, Shaanxi, People’s Republic of China; Eberhard-Karls University, Germany

## Abstract

**Introduction:**

Urethral stricture, a frequent source of lower urinary tract disorders in men, is still a difficult problem for urologists. Based the anti-restenosis effect of paclitaxel on coronary artery, the role of docetaxel, a semi-synthetic analogue of paclitaxel, in limiting urethral stricture formation was studied.

**Methods:**

Forty adult New Zealand male rabbits were involved in this study, which were randomly assigned into 3 groups, namely a high dose docetaxel (D_H_, 0.1 mg/d), a low dose docetaxel (D_L_, 0.01 mg/d) and a control (C) group, with 16, 16, 8 rabbits in each group, respectively. All animals underwent a 10 mm-long circumferential electrocoagulation of the bulbar urethra with a 13Fr pediatric resectoscope. Drugs were given by urethral irrigation daily and continuous for 28 days. Stricture formation was assessed by retrograde urethrography and videourethroscopy. Urethra pathology was evaluated by hematoxylin and eosin staining and Sirius red staining.

**Results:**

At the end of this study, 15, 14 and 7 rabbits remained for evaluation in D_H_, D_L_ and C group, respectively. Urethral diameters in D_H_, D_L_ and C group were (7.17±1.63) mm, (6.55±0.62) mm, (3.23±1.36) mm, with a normal urethral diameter of (9.08±1.29) mm. Lumen reduction in D_H_, D_L_ and C group were (36.93±11.58)%, (48.03±7.89)% and (84.66±14.95)%, respectively. Statistically difference could be found between every two groups (*p*<0.05) both in urethral diameters and in lumen reduction, except for compare of urethral diameters between D_H_ and D_L_ group. Histological examination confirmed mass fibrous tissue and collagen content at the stricture sit in C group, whereas less in docetaxel treated rabbits.

**Conclusions:**

Docetaxel could limit urethral stricture formation, which may be due to inhibition of fibrous tissue and collagen expression. Docetaxel may become a new choice in the prevention of urethral stricture formation.

## Introduction

Urethral stricture is a frequent source of lower urinary tract disorders in men, characterized by narrowing of the urethra by a noncompliant section of urethral scar tissue. Currently the most common causes of urethral stricture are idiopathic, transurethral resection, urethral catheterization, pelvic fracture and hypospadias-surgery [1]. Urethral stricture is commonly treated with urethral dilation and visual internal urethrotomy, which can transiently improve urinary flow. Nevertheless, repeat instrumentation exacerbates scar formation complicating subsequent reconstruction [2], and repeated dilation or urethrotomy are neither clinically effective nor cost effective [3]. While urethroplasty offers a better cure rate [4], but it is not a routine operation and requires a certain expertise.

The pathogenesis of urethral stricture is a process of fibrosis formation, caused by excessive collagen synthesis and changes in the composition of the extracellular matrix. Varying degrees of spongiofibrosis results in poorly compliant tissue and decreased urethral lumen caliber. Enlightened by the treatment of organ fibrosis, a few antifibrotic drugs were being used in trial of limiting urethral stricture formation, such as halofuginone, mitomycin C, bitoxin A, somatostatin analogue, glucocorticoid [5], [6], [7], [8], [9]. In the near decades, it is widely confirmed that rapamycin and paclitaxel eluting stent showed a satisfactory inhibiting effect on restenosis of coronary artery [10], [11]. And our previous study had exposed rapamycin could inhibit urethral stricture formation in rabbits [12]. In this study, the role of docetaxel, a semi-synthetic analogue of paclitaxel, on inhibition of urethral stricture formation was investigated.

## Materials and Methods

### 2.1 Ethics Statement

This study was carried out in strict accordance with the recommendations in the Guide for the Care and Use of Laboratory Animals of the National Institutes of Health. The protocol was approved by the Committee on the Ethics of Animal Experiments of Xi’an Jiaotong University.

### 2.2 Animals and drugs

Totally 40 adult male New Zealand rabbits, aged 4 months and weighing (2.0±0.2) kg, were involved in this experiment, which were randomly allocated into 3 groups, namely a high dose docetaxel (D_H_), a low dose docetaxel (D_L_) and a control (C) group, with 16, 16, 8 rabbits in each group, respectively. The rabbits were kept in special cages in SPF environment, allowing free movement and free access to food and drink. Animals were raised in natural solar day-night cycles, fed with a special kind of solid fodder produced by Laboratory Animal Center of Xi’an Jiaotong University.

All process, including surgery, radiology, urethroscopy and sacrifice, was performed under diazepam and ketamine anesthesia, and all effort were made to minimize suffering. Docetaxel was gifted by Hengrui Pharmaceutical Company. ltd. Jiangsu province.

### 2.3 Production of urethral strictures

All rabbits were anaesthetized by premedication with i.v. diazepam (25 mg/kg) and i.v. Ketamine (2.5 mg/kg). For endoscopic procedures a 13Fr pediatric resectoscope (Hangzhou Hawk Optical Electronic Instruments Co., Ltd) was used. The rabbits were placed in a supine position. A 10 mm long circumferential electrocoagulation of the bulbar urethra was induced under sterile conditions. This procedure was performed distal to the verumontanum and always away from the external sphincter with a loop-shaped electrode at a power of 40 W [13]. Electrocoagulation was continued until blanching and ulceration of the mucosa occurred [13], [14] All electrocoagulation were done by the same urologist (Hecheng Li). The urine was not diverted deliberately and no antibiotics were given.

### 2.4 Drug administration

Drugs were given by retrograde urethral irrigation, once a day and last 28 days since the first day after electrocoagulation. The administration doses in D_H_, D_L_ were 0.1 mg/day and 0.01 mg/day respectively and the irrigating volume was 10 ml every time for each animal. For rabbits in C group, 10 ml normal saline was injected instead.

### 2.5 Evaluation of urethral strictures

Twenty-eight days after electrocoagulation, to evaluate formation of urethral restriction, urethrograms and video-urethroscopy were used to measure the caliber of urethra on anesthetized animals. As described by Chong [12], meglumine amine (380 g/L) was used as contrast medium, which was slowly and directly injected into the urethra under X-ray monitoring. Retrograde urethrography was continued until urethral lumen was manifested well. All process was done by the same urologist (Delai Fu). Referring to Meria [14], urethra 1 cm distal to the injured site was regarded as normal. Diameters of injured urethra and normal urethra were measured blindly. Urethral diameters and lumen reduction were compared among groups. Strictures were considered significant if the urethral lumen was decreased more than 50%. Video-urethroscopy was performed with a 13Fr pediatric resectoscope to further evaluate urethral stricture formation.

### 2.6 Histological evaluation

Rabbits were killed under anesthesia by intravenously injection 50 ml of air. The urethras from all rabbits were processed for histological examination as described by Anderson [15]. Initial tissue fixation was done before urethra was moved. Formaldehyde was injected into the urethra under a pressure of 1.8 kPa for 30 min. This was carried out by a special device, composed of a flexible pipe and a container. The pipe linked the orifice of urethra with the container. The container maintained liquid level of 18 cm above the operative platform, producing a pressure of 1.8 kPa. Then urethra was removed, further fixed in formaldehyde for 24 hours and embedded in paraffin. Sections were cut corresponding to the two distension sites longitudinally from the posterior wall of the urethra, perpendicularly to the wall, and stained with HE and sirius red. All slides were blindly reviewed by the same uropathologist.

### 2.7 Statistical analysis

Results of urethral diameter and lumen reduction are expressed as Mean ± SD. The amount of urethral fibrosis was evaluated as mild, moderate or severe. The One-Way ANOVA test was used for statistical analysis. A *p* value less than 0.05 was considered statistically significant.

## Results

One rabbit in D_H_ group, two rabbits in D_L_ and one in C group died during anesthesia. Urethral bleeding was seen in almost all rabbits after electrocoagulation, but it was not severe and totally disappeared in 3 days. Thus, 15, 14 and 7 rabbits remained for evaluation in D_H_, D_L_ and C group, respectively. No urinary retention occurred till the end of the study.

### 3.1 Formation of urethral stricture

Urethral diameters in D_H_, D_L_ and C group were (7.17±1.63) mm, (6.55±0.62) mm, (3.23±1.36) mm, respectively, with a normal urethral diameter of (9.08±1.29) mm on the average. Statistically difference could be found between every two groups (*p*<0.01), except for compare of urethral diameters in D_H_ and D_L_ group (*p* = 0.218)_._

**Figure 1:**
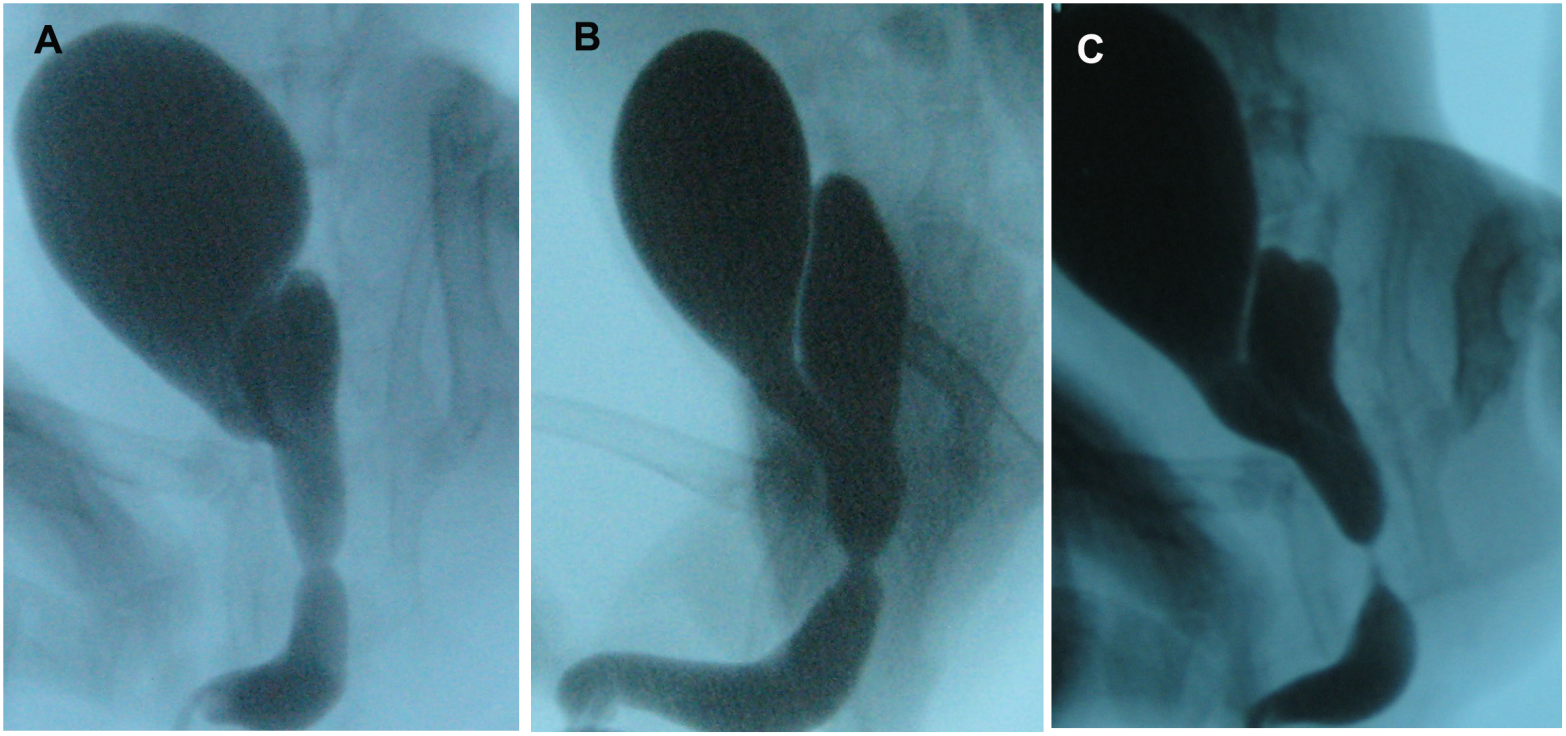
Representative urethral stricture formation in retrograde urethrogram. A Urethral stricture formation in rabbits of D_H_ group. Note mild stricture formation at bulbar urethra. B Urethral stricture formation in rabbits of D_L_ group. Note moderate stricture formation at bulbar urethra. C Urethral stricture formation in rabbits of C group. Note severe stricture formation at bulbar urethra.

According to urethral lumen reduction, all rabbits in C group had significant urethral stricture formation (lumen reduction >50%), while 6 (37.5%) in D_L_ group and none (0%) in D_H_ group. The lumen cross section area decreased (84.66±14.95)% (rang, 60.94–96.48%) in C group, (48.03±7.89)% (rang, 34.22–62.00%) in D_L_ group and (36.88±11.58)% (rang, 10.71–48.65%) in the D_H_ group. Urethral lumen reduction was significantly more severe in C group than in the D_H_ group (*p*<0.01) and in the D_L_ group (*p*<0.01). Lumen reduction was statistically more in the D_L_ group than in the D_H_ group (*p* = 0.010, Fig. 1, Table 1).

**Table 1:** Urethral diameter and lumen reduction of rabbits in different treated groups.

Group	n	Urethral diameter (mm, mean ± SD)	Lumen reduction (%)
D_H_	15	7.17±1.63*	36.93±11.58#
D_L_	14	6.55±0.62**	48.03±7.89##
C	7	3.23±1.36***	84.66±14.95
Normal•	36	9.08±1.29	

•Normal referred to diameter of urethra 1 cm distal to the injured site of all rabbits in this study.

*P = 0.218 compared to D_L_, *P*<0.01 compared to C and Normal.

**P<0.01 compared to C and Normal.

***P<0.01 compared to Normal.

# *P* = 0.010 compared to D_L_, *P*<0.01 compared to C.

## *P*<0.01 compared to C.

**Figure 2:**
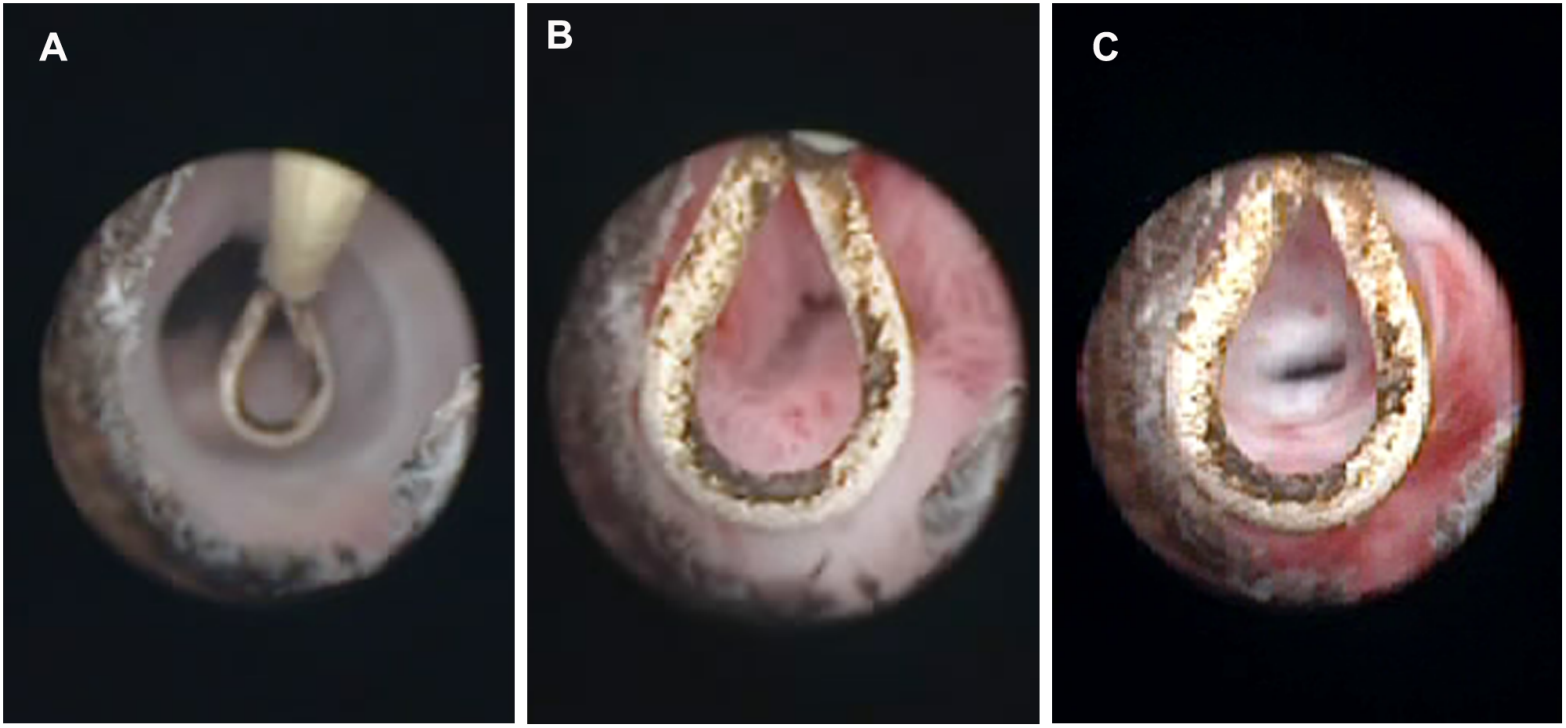
Urethral stricture formation under urethroscopy. A Urethroscopy of rabbit in D_H_ group. Note no significant urethral stricture formation and F13 urethroscopy could easily pass through. B Urethroscopy of rabbit in D_L_ group. Note urethral stricture formation and F13 urethroscopy could hardly pass through. C Urethroscopy of rabbit in C group. Note severe urethral stricture formation, stopping F13 urethroscopy pass through.

Based on urethroscopy, varying degree of bulbar urethra narrowing was found and the injured urethral surface had been covered by epithelium in all rabbits (Fig. 2).

### 3.2 Urethral histology

**Figure 3:**
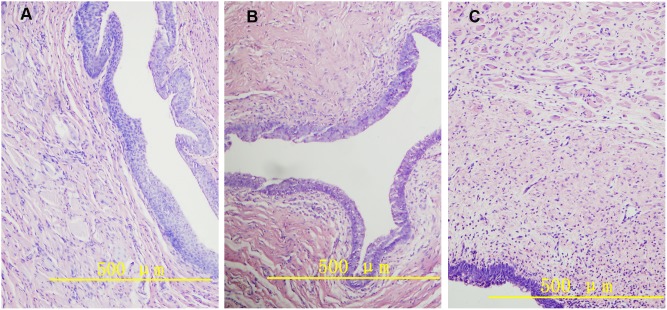
Urethral cross section at stricture sites (hematoxylin and eosin staining) Original magnifications, 400×. A Urethral cross section of rabbit in D_H_ group. Note little fibrosis tissue around urethral epithelium. B Urethral cross section of rabbit in D_L_ group. Note fibrosis formation around submucosa layer. C Urethral cross section of rabbit in C group. Note wide fibrous tissues distributed from epithelium to muscular layer.

**Figure 4:**
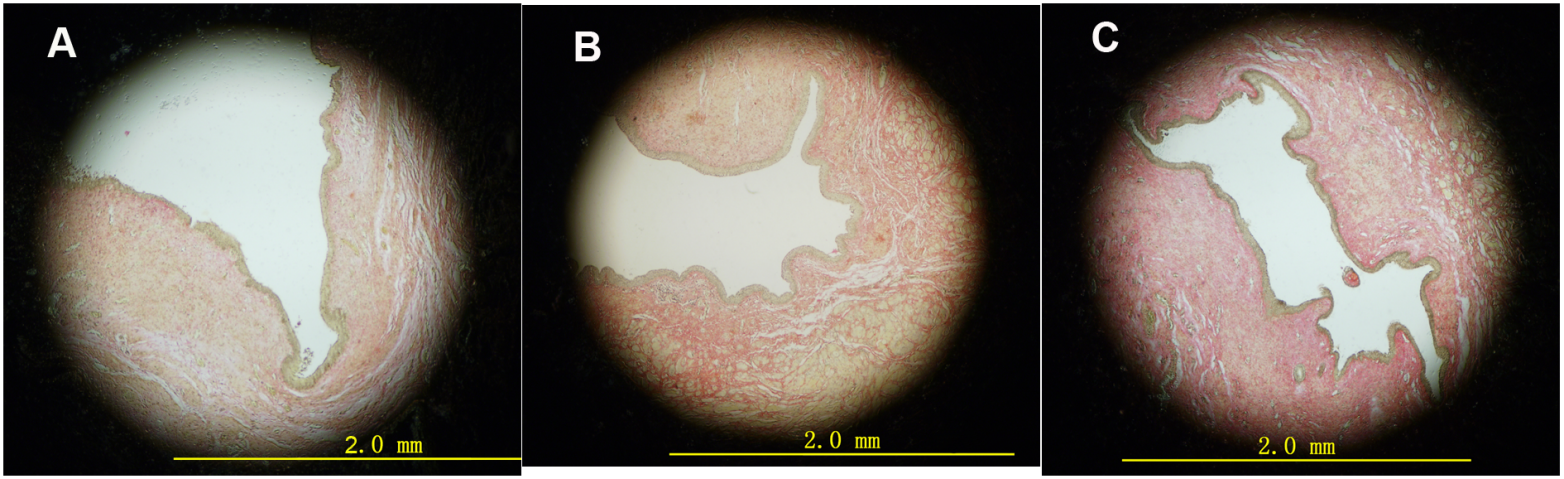
Representative microphotographs of urethral cross section at stricture site (stained by Sirius red). A Urethral cross section of rabbit in D_H_ group. Note a large urethral lumen with light submucosa collagen staining. B Urethral cross section of rabbit in D_L_ group. Note large urethral lumen with light submucosa collagen staining. C Urethral cross section of rabbit in C group. Note almost atresic urethra with deep collagen staining.

With H&E staining, a varying degree of fibrosis was found beneath the epithelium in differentially treated rabbits. For animals in D_H_ group and D_L_ group, this fibrosis was mainly localized in submucosa layer, while in control group, fibrosis distributed over submucosa and muscular layer (Fig. 3). Sirius Red staining showed light red staining of the urethral stricture site in D_H_ and D_L_ group, indicating relatively low collagen concentration. However, darker crimson staining was found in C group, indicating abundant collagen presence (Fig. 4).

## Discussions

The present study was undertaken to investigate the effect of intraurethral injection of docetaxel on urethral stricture formation in rabbits. Our preliminary results demonstrated that docetaxel could significantly inhibit urethral stricture formation. Urethral diameter in C group decreased to (3.23±1.36) mm from (9.08±1.29) mm in normal. Docetaxel inhibits this process with calibers of (7.17±1.63) mm and (6.55±0.62) mm in D_H_ and D_L_ group, respectively. Urethral lumen reduction in D_H_ and D_L_ group decreased to less than 50%, while that in C group decreased more than 80%. The effect of docetaxel on inhibiting urethral stricture formation may mediate by inhibition of fibrosis formation and collagen coagulation. Although there are limitations to this pilot study, our results firstly suggest that intraurethral irrigation of docetaxel could obviously inhibit urethral stricture formation in rabbits.

Additionally and interestingly, no difference of urethral diameter was found between rabbits in D_H_ and D_L_ group (*p* = 0.218), while judged with urethral cross-sectional area change, less lumen reduction in D_H_ group than in D_L_ group could be detected (*p* = 0.010). A possible explanation of this distinction is the limited animal number and the deviation of urethral diameter between rabbits. Lumen reduction seems to be more sensitive than diameter in the evaluation of urethral stricture. A dose-dependent manner of docetaxel on inhibiting urethral stricture formation should and would appear with the increase of rabbit number.

Recent studies have shown that paclitaxel possess inhibitory effect on fibrosis diseases. In 2010, Zhang [16] et al first reported that intraperitoneal injection of low-dose paclitaxel (0.3 mg/kg, twice a week, sustained for 7 or 14 days) could reduce renal tubulointerstitial fibrosis in unilateral ureteral obstruction model in rat. At the same time, Zhou [17] et al cultured hepatic stellate cells in rat (RHSCs) and found paclitaxel, with a concentration of 200 nmol/L, could inhibit TGF-β induced RHSCs fibrosis. One year later, Sun [18] et al used the same dose and route of paclitaxel administration as Zhang [16] and indicated paclitaxel could ameliorate renal fibrosis with improvement in renal functions in a rat model of remnant kidney disease. A paclitaxel-eluting stent was even used in prevention of human malignant biliary obstruction [19]. Nevertheless, it was reported that prolonged chemotherapeutic treatment with paclitaxel/docetaxel was associated with canalicular stenosis, pulmonary fibrosis or scleroderma-like changes, albeit in only a small fraction of the patients [20], [21], [22]. It should be indicated that the inhibition of tumor cell proliferation can be achieved by much higher dosages of paclitaxel. However, according to Zhang [16], the inhibition of fibrosis formation can be attained with very low dose of paclitaxel. This may explain the contradictory effect of paclitaxel/docetaxel on fibrosis formation.

Docetaxel, as a semi-synthetic analogue of paclitaxel, is two to three times as effective as paclitaxel in promoting the assembly of mammalian brain tubulin in vitro and has a binding constant that is greater than that of paclitaxel by the same factor [23]. As a result, in this study docetaxel was used instead of paclitaxel.

As an anticancer agent, docetaxel could stabilize polymerized microtubules and enhance microtubule assembly, consequently arrests the cell cycle in the G0/G1and G2/M phases, leading to cell death. It has been previously shown that microtubules could bind endogenous Smad2 and Smad3 and form a complex. Destabilization of the microtubules network by nocodazole, olchicine, or a tubulin mutant disrupts the complex between Smads and microtubules. TGF-β triggers dissociation of Smad2 and Smad3 form microtubules, leading phosphorylation and nuclear translocation of Smad2 and Smad3, with consequent activation of transcription, increasing production of extracellular matrix components. So microtubules serve as a cytoplasmic sequestering network for Smads, controlling Smads association with and phosphorylation by activated TGF-β receptor I [24]. Docetaxel stabilizes microtubules and inhibits microtubules depolymerization, suppressing Smad2 and Smad3 phosphorylation and collagen deposition [25]. That may be why docetaxel works in limiting urethral stricture formation.

In this pilot study in rabbits urethral stricture model, dose rates (0.1 versus 0.01 mg/day) of docetaxel were designed based on the reports of Zhang [16] and Sun [18] and the fact that rabbits are approximately one-half tolerant of docetaxel than rats. Considering minimal absorption by the mean of intraurethral irrigation, docetaxel was given daily. These dose rates are quite lower than used in anticancer chemotherapy. Meanwhile there is evidence to show that low-dose docetaxel had minimal, if any, detectable effects on cell proliferation [16]. According to the above analysis, we have reason to establish the assumptions that low-dose docetaxel inhibits urethral stricture formation may not via inhibition of cell proliferation, such as fibroblasts, but due to interruption on TGF-β signaling pathway. More studies are needed to confirm this hypothesis.

## Conclusions

In this rabbit model, docetaxel, given by intraurethral irrigation was effective in limiting the occurrence of de novo urethral stricture. Docetaxel may become an important therapy for urethral stricture in humans. However, further studies including a larger number of animals with a longer follow up and containing more details about TGF-β/Smads signaling are needed to consolidate these conclusions.
